# Knowledge, Self-Confidence and Attitudes towards Suicidal Patients at Emergency and Psychiatric Departments: A Randomised Controlled Trial of the Effects of an Educational Poster Campaign

**DOI:** 10.3390/ijerph14030304

**Published:** 2017-03-14

**Authors:** Renate van Landschoot, Gwendolyn Portzky, Kees van Heeringen

**Affiliations:** Unit for Suicide Research, Department of Psychiatry and Medical Psychology, Ghent University, 9000 Ghent, Belgium; gwendolyn.portzky@ugent.be (G.P.); cornelis.vanheeringen@UGent.be (K.v.H.)

**Keywords:** suicide prevention, educational poster campaign, knowledge, self-confidence, attitudes

## Abstract

Educational posters are used to enhance knowledge, attitudes and self-confidence of patients. Little is known on their effectiveness for educating health care professionals. As these professionals may play an important role in suicide prevention, the effects of a poster and accompanying evaluation and triage guide on knowledge, self-confidence and attitudes regarding suicidal thoughts and behaviours, were studied in a multicentre cluster randomised controlled trial, involving staff from 39 emergency and 38 psychiatric departments throughout Flanders (*n* = 1171). Structured self-report questionnaires assessed the knowledge, confidence and beliefs regarding suicidal behaviour management, and attitudes. Data were analysed through a Solomon four-group design, with random assignment to the different conditions. Baseline scores for knowledge and provider confidence were high. The poster and accompanying evaluation and triage guide did not have an effect on knowledge about suicide and self-confidence in suicidal behaviour management. However, the poster campaign appeared to be beneficial for attitudes towards suicidal patients, but only among staff from mental health departments that were assigned to the un-pretested condition. Given the limited effects of the poster campaign in the studied population with a relatively high baseline knowledge, the evaluation of this poster as part of a multimodal educational programme in a more heterogeneous sample of health care professionals is recommended.

## 1. Introduction

Suicide is a major global public health problem accounting for more than 800,000 deaths each year. The annual mortality rate is estimated at 11.5 deaths per 100,000 people, which equates to one death every 40 [1].

Accumulating evidence shows that training and educating gatekeepers is a worthwhile investment in suicide prevention. Gatekeepers may play a pivotal role in the early identification, management and referral of suicidal patients [1,2,3,4]. They can be among the first to screen and intervene for suicide risk as they may be in close contact with suicidal individuals and therefore have the opportunity to interrupt an ongoing suicidal process [5,6]. Front-line health professionals, such as general practitioners, mental health professionals and emergency department staff, report that targeted education and training in suicide prevention would be helpful [3,7,8,9,10]. Furthermore, a broad range of both health and community professionals appear to benefit from education and training interventions [3,11,12,13,14]. In particular, such interventions may provide gatekeepers with better knowledge about suicide, promote more adaptive attitudes towards suicidal patients, and increase provider confidence in assessing and managing suicide risk [15,16,17,18,19,20,21,22].

The field of suicide prevention has made great strides in developing education and training interventions for various key groups, e.g., gatekeeper training, workshops with actors role-playing patients, e-learning, 1-day train-the-trainer programmes, and educational posters campaigns [2,23,24,25,26]. Educational poster campaigns have been used for a long time in various health domains. Targeting a wide range of health promotion issues they may well be a promising tool to raise awareness, increase knowledge and elicit behaviour change among patients [27,28].

Educating health professionals about suicide prevention is a component of many national suicide prevention strategies, but the effects of educational poster campaigns regarding early detection of suicide risk, intervention, follow-up and referral of suicidal patients have hardly been studied. Currier et al. [3]) suggested that an educational poster and accompanying evaluation triage guide may be a simple and cost-effective tool for emergency department staff in increasing provider awareness and improving provider perception of knowledge and skills regarding the identification and management of suicidality. However, the interpretation of the findings of this study is hampered by methodological shortcomings including the use of non-validated measurements and the inclusion of only one comparator site.

Therefore, the current study aimed at developing and evaluating a poster campaign using validated measurements and multiple controls. It was hypothesized that the poster and accompanying evaluation and triage guide will improve knowledge regarding suicidality, will increase provider confidence in assessing and treating at-risk individuals, and will lead to more adaptive attitudes towards suicidal patients for staff of both emergency and psychiatric departments in Flanders.

## 2. Materials and Methods

### 2.1. The Poster Campaign

We used a Flemish adaptation of the “Is Your Patient Suicidal?” poster that was originally developed by the Suicide Prevention Resource Center (SPRC) in the United States [3]. This is a four-color A3 size poster entitled “Is Your Patient Suicidal?”, which is accompanied by a 1-page, double-sided clinical triage guide. The poster offers information on identifying and responding to high-risk patients, including (1) the most common and manifest signs of acute suicide risk; (2) facts and figures; (3) questions that can be used to detect and discuss suicidal ideation and attempt history when signs are noticed of suspected; and (4) referral for additional suicide prevention services. The accompanying guide “Suicide risk: A guide for evaluation and triage” provides further guidance to identify suicidal ideation and suicidal intent, triage criteria to evaluate the level of risk (including interventions concerning high-risk patients, moderate-risk patients and low-risk patients), and checklists for discharge and documentation.

The educational materials were adapted to the Flemish context of this trial and field-tested in different focus groups, including the Flemish task force of suicide prevention and clinical staff of emergency and psychiatric departments. The poster and guide were displayed for four weeks in strategic staff-only sites such as meeting rooms, lunchrooms and staff toilets.

### 2.2. Subjects

Emergency and psychiatric departments were recruited from July 2013 until January 2014. In total, 49 Flemish hospitals agreed to participate, accounting for 64.5% of the total number of hospitals in the Flanders region. At the individual level, 2364 health professionals from emergency and psychiatric departments throughout Flanders were invited to participate, of whom 1171 (49.5%) agreed to participate. The study population included 638 (54.5%) emergency department (ED) staff and 533 (45.5%) mental health professionals.

In order to evaluate the impact of the educational poster campaign on knowledge, confidence and attitudes of staff of these two types of hospital departments, a Solomon four-group design was used. This design allows for the control of pretesting effects by including both experimental and control conditions with and without initial pretesting [29]. Therefore, the subjects of the two department types were randomly assigned to one of the following four groups: (1) one experimental group of health professionals being assessed before and after exposure to the poster campaign (*n* = 212; 14 departments); (2) one control group of health professionals being assessed twice over a time frame comparable to the experimental group (*n* = 338; 22 departments); (3) one experimental group of health professionals being assessed only after exposure to the poster campaign (*n* = 298; 21 departments); and (4) one control group of health professionals assessed only once (*n* = 323; 18 departments).

As the poster campaign was conducted at the department level, and not at the individual level, a cluster design was adopted with department being the unit of randomisation. In order to fulfil the sample size requirements, no restrictions on cluster size were imposed to the hospital departments. Since some hospital departments covered over 60 potential subjects, while others only identified 15 eligible participants, cluster sizes vary. Consequently, there was a slight difference in the numbers of subjects of the two hospital departments assigned to each condition.

### 2.3. Sample Size

As the power of a Solomon four-group design is supposed to be greater than that of a post-test-only control group design [30], the power was calculated based on a two-sample *t*-test on the post-test-only groups. To account for the cluster design, an intra-cluster coefficient (ICC) of 0.05 was assumed. As no values for ICC under this setting were available in the literature, an assumption was made based on general practice and medical trials in which ICC values were reported between 0.01 and 0.05 [31,32]. In order to demonstrate an effect-size of 0.4 between the intervention groups (1 and 3) and the control groups (2 and 4) with a power of 80%, a statistical significance of 5% and an ICC of 0.05, 16 departments of at least 15 health professionals were needed in both the intervention groups (1 and 3) and the control groups (2 and 4).

### 2.4. Inclusion Criteria

Staff of both emergency and psychiatric departments in Flanders were eligible to participate in the study if they (a) were 18 years or older; (b) were in close contact with suicidal patients (e.g., physicians, physician assistants, psychiatrists, psychologists, and nurses) and (c) provided informed consent to participate.

### 2.5. Exclusion Criteria

Non-clinical hospital staff (e.g., administrative personnel, and ambulance drivers) were not eligible for participation. Study coordinators of participating departments were intensively informed about the educational intervention. In order to avoid bias of results, they were excluded from participating in the study.

### 2.6. Procedure

Ethical approval was obtained from the ethical board of the University Hospital of Ghent in accordance with the ethical principles expressed in the Declaration of Helsinki (ethical approval code EC/2013/473). Given the multicentre character of the study, the study protocol was also approved by the institutional review board of each participating site.

Consistent with the study procedure of the SPRC, the study involved three phases including (1) completion and collection of baseline questionnaires (lasting 3 weeks; Questionnaire S1); (2) exposure to the educational poster campaign (displayed for 4 weeks); and (3) completion and collection of follow-up questionnaires (lasting 3 weeks).

The study coordinators (mostly head nurses) of the departments were asked to facilitate the study by distributing the paper-and-pencil surveys during regular staff meetings and encouraging personnel to participate. In order to match baseline and follow-up questionnaires without compromising the confidentiality of staffs’ responses, each participant created his or her own unique identification number.

### 2.7. Measures

After giving informed consent, clinical hospital staff anonymously completed self-reports, covering sociodemographics and the following questionnaires.

A subscale of the Dutch translation [33] of the 14-item Question, Persuade and Refer questionnaire (QPR) was used to assess self-perceived knowledge about suicide [34]. Levels of knowledge were assessed using questions such as ‘How do you rate your knowledge about suicide warning signs?’. Answers were given on a Likert scale ranging from 1 (very low) to 5 (very high). Responses were summed to provide a total score ranging from 7 to 35, with higher scores representing greater levels of self-perceived knowledge. The QPR has been shown to reliably assess effects of training on self-perceived knowledge of suicide prevention [23,35,36].

The eight items of the Suicide Information Test (SIT) asking about warning signs and risk factors [37] was used to assess knowledge about suicide more objectively. The original questionnaire is comprised of 28 true-false items and was translated into Dutch and adjusted for a Flemish randomized controlled trial [38]. The questionnaire includes statements such as ‘Suicidal tendencies are inherited, and suicide runs in families’. Clinical staff could agree (score 1) or disagree (score 0) with the eight statements, resulting in total scores ranging from 0 (disagreed with all statements) to 8 (agreed with all statements). Higher scores thus reflect greater knowledge about warning signs and risk factors of suicide.

A subscale of the Confidence and Beliefs Questions (CBQ) was used to measure provider confidence in suicidal behaviour management [22]. The questionnaire was translated into Dutch for the clinical trial of De Beurs and colleagues [33]. The subscale consists of three items. Example: ‘I am confident in my ability to successfully treat a suicidal patient’. Scoring occurs on a 5-point Likert scale ranging from ‘strongly disagree’ to ‘strongly agree’. The subscale is summed to derive a total subscore ranging from 3 to 15, with higher scores indicating higher levels of provider confidence. The CBQ has been found to measure differences in confidence regarding suicide care [22]. 

Attitudes towards suicidal behaviour and suicidal patients were assessed using an adjusted version of the Attitudes Towards Suicide Questionnaire (ATTS) [39]. The original 37-item instrument was translated into Dutch and was reduced to 29 items on the basis of a confirmatory factor analysis reported by De Clerck and colleagues [40]. In this study, only the three items concerning the factor willingness to help were considered relevant. Example: ‘It is a humane duty to try to stop someone from dying by suicide’. Responses are scored on a 5-point Likert scale from 1 (completely disagree) to 5 (completely agree). A sum score was calculated from 3 to 15, with higher scores reflecting more adaptive attitudes, i.e., greater willingness to help suicidal patients. The ATTS is a valid and reliable measure in clinical and community samples for determining attitudes towards suicidal behaviour, demonstrating high internal consistency and test-retest reliability [39,41,42].

### 2.8. Study Design

This study was a multicentre clustered randomized controlled trial that was conducted at emergency and psychiatric departments in Flanders. Departments were randomized to one of the four conditions based on a block design (4 per block) and stratified by department type using random allocation software. Main analyses were conducted separately for staff of emergency and psychiatric departments. The methods used are those described in ‘Statistical treatment of the Solomon four-group design: a meta-analytic approach’ [30]. However, they were adapted to the clustered design by applying mixed models with cluster (combination of hospital and department) as a random effect.

Preliminary analyses included chi-square analysis and *t*-tests to assess baseline differences between the pretested conditions. The initial phase of the analysis associated with the Solomon four-group design, started with a 2 × 2 between-groups analysis of variance (ANOVA) on the post-test scores, with the two main effects being pre-test vs. no pre-test and intervention vs. no intervention. If no significant outcomes were observed for both the main effect of pre-test and the interaction of pre-test by poster campaign, the data from the two pretested groups were reanalysed by using a two-group analysis of covariance (ANCOVA) with pre-test scores as control variables (covariate) and post-test scores as criterion variables (dependent variables). SPSS version 22 (Chicago, IL, USA) was used for all analyses. Data were presented as means with 95% confidence intervals (CI). The level of significance was set at *p* < 0.05.

## 3. Results

### 3.1. Baseline Results

Table 1 shows the baseline characteristics for the experimental and the control group at the department level and the staff level. At the department level, there were no differences in the number of emergency and psychiatric departments assigned to each pretested condition (7 and 11 respectively).

The baseline sample consisted of 307 (55.8%) mental health professionals and 243 (44.2%) ED staff. The majority were women (*n* = 358, 65.1%). All age groups between 18 and 65 years were well represented, with a mean age of 38.25 years (SD = 11.23). Participants comprised predominantly general nurses (*n* = 269, 48.9%) and psychiatric nurses (*n* = 135, 24.5%). Years of professional experience as ED or mental health care provider ranged from 0 years to 40 years (M = 14.5 years, SD = 10.6). Frequency of contact with suicidal patients varied according to department, with 64.2% of mental health professionals reporting daily contact with suicidal patients compared to 16.1% of ED providers (χ^2^(1) = 118.436, *p* < 0.001). About half (54.9%) reported having additional education or training in suicide prevention within 6 months prior to the baseline assessment of this study, with significant differences between staff of psychiatric and emergency departments (70.8% vs. 35.0% respectively; χ^2^(1) = 63.878, *p* < 0.001). A significant amount of health professionals reported experience with suicide or suicidal behaviour in their personal environment, i.e., a family member (*n* = 100, 37.0%), close friend (*n* = 141, 31.2%), colleague or acquaintance (*n* = 227, 47.6%).

Analysis regarding the subscale of the QPR questionnaire revealed a mean score of 24.1 (SD = 3.8; Min = 7, Max = 35). Almost half of the baseline sample (*n* = 241, 43.8%) rated their general understanding about suicide and suicide prevention as ‘high’ or ‘very high’. Significant differences in self-perceived knowledge level were found between staff of psychiatric and emergency departments, with mental health professionals reporting higher baseline knowledge scores than ED providers (M = 25.2 vs. M = 21.6; t(507) = −12.42, *p* < 0.001).

Analysis regarding the SIT showed that baseline levels of knowledge regarding risk factors and warning signs of suicide were considerably elevated. With regard to the 3 items asking about risk factors, 81.6% of the health professionals answered at least 2 items correctly. Similar results were found for the 5 items asking about warning signs, with 75.5% of the subjects answering at least 4 items correctly. There were no significant differences in level of knowledge regarding risk factors between staff of emergency and psychiatric departments (M = 2.1 vs. M = 2.1; t(470) = −0.026, *p* = 0.98). However, mental health professionals appeared to have greater knowledge about warning signs than ED providers (M = 4.1 vs. M = 3.8; t(468) = −4.019, *p* < 0.001).

With regard to attitudes, the mean ATTS subscore was 12.4 (SD = 2.0; Min = 6, Max = 15) indicating that baseline attitudes of clinical staff are quite adaptive. However, mental health professionals and ED staff significantly differed in their willingness to help suicidal patients (M = 12.8 vs. M = 11.8; t(510) = −5.695, *p* < 0.001). Staff of psychiatric departments more readily endorsed that ‘they are prepared to help a person in s suicidal crisis by making contact’ (93.5% vs. 76.9%; χ^2^(1) = 29.141, *p* < 0.001) and reported more disagreement with the statement ‘if someone wants to commit suicide, it is their business and we should not interfere’ (85.9% vs. 77.0%; χ2(1) = 29.141, *p* = 0.009).

At baseline, the vast majority of clinical staff reported no hesitancy in asking about patients’ current suicidal ideation (*n* = 390, 76.5%) and about half reported feeling confident or very confident in its ability to successfully assess (*n* = 282, 54.8%) and treat (*n* = 256, 49.9%) suicidal patients. There were significant differences in provider confidence among staff of emergency and psychiatric departments. ED providers were less confident in the assessment and treatment of suicidal behaviour and were more hesitant to ask a patient if he or she is suicidal compared to mental health professionals (M = 10.0 vs. M = 11.7; t(507) = −9.581, *p* < 0.001).

There was no statistically significant difference between the two pretested conditions in terms of demographics and outcome measures at baseline.

### 3.2. Posttest Results

#### 3.2.1. Self-Perceived Knowledge

First, a 2 × 2 mixed analysis of variance (ANOVA) was conducted on the four total post-test scores of the QPR questionnaire. The two factors were pre-test (yes vs. no) and poster campaign (yes vs. no). For both staff of emergency and psychiatric departments, the ANOVA showed no significant interaction effect of pre-test by poster campaign (F = (1, 44) = 1.463, *p* = 0.23; F = (1, 28) = 0.164, *p* = 0.69, respectively). As an interaction effect could not be identified (referred to as ‘Test A’) [30], an examination of the main effect of poster campaign followed (referred to as ‘Test D’) [30]. For ED providers as well as mental health professionals, this main effect was not significant (F = (1, 34) = 0.306, *p* = 0.58; F = (1, 28) = 0.818, *p* = 0.37 respectively). Furthermore, a two-group analysis of covariance (ANCOVA) was performed on the total post-test scores, covarying the total pre-test scores (referred to as ‘Test E’) [30]. “Test E is the preferred test of these three (Tests E, F, and G), however, primarily because of its greater power or ability to detect the treatment effect” [30] (p. 151). For staff of both departments, the ANCOVA showed no significant effects of poster campaign in Condition 1 and 2, the two pretested groups (F = (1, 7) = 0.117, *p* = 0.74; F = (1, 14) = 0.199, *p* = 0.66, respectively). Because significance of the ANCOVA was lacking, a *t*-test was performed on the scores of Condition 3 and 4, the post-test only groups (referred to as ‘Test H’) [30]. Again, for both staff of emergency and psychiatric departments the results of the *t*-test were not significant (t(19) = −1.14, *p* = 0.27; t(13) = 0.38, *p* = 0.71 respectively). Finally, the results of Test E and H were combined with a meta-analysis (referred to as ‘Test I’) [30]. For both ED and mental health providers, the meta-analysis was not significant (zmeta = 1.01, *p* = 0.31; zmeta = 0.57, *p* = 0.57, respectively).

#### 3.2.2. Actual Knowledge

For staff of both emergency and psychiatric departments, no significant results were found for the SIT scores.

#### 3.2.3. Provider Confidence

For staff of both emergency and psychiatric departments, the results of the analyses of the CBQ scores did not achieve levels of significance.

#### 3.2.4. Attitudes

For ED providers, a 2 × 2 ANOVA on the four ATTS post-test scores revealed that the interaction effect of pre-test by poster campaign was substantial but not significant by conventional standards (F = (1, 50) = 3.130, *p* = 0.08). Subsequent ANOVA could not identify a significant main effect of the poster campaign on attitude (F = (1, 40) = 1.936, *p* = 0.17). In addition, the ANCOVA was not significant (F = (1, 9) = 0.123, *p* = 0.50). The *t*-test that was performed on the scores of the post-test only groups, seemed not to be significant (t(17) = −0.15, *p* = 0.88). The meta-analysis was also not significant (zmeta = 0.58, *p* = 0.56). Among mental health professionals, the ANOVA analysis could, however, reveal a significant interaction between pre-test and poster campaign (F = (1, 27) = 6.139, *p* = 0.02). Therefore, a main effects analysis was performed on the pretested groups (referred to as ‘Test B’) [30] but no significant simple effect of the poster campaign could be identified (F = (1, 9) = 0.492, *p* = 0.50). Subsequently, a simple main effects test was conducted on the post-test only groups (referred to as ‘Test C’) [30]. This result was significant, indicating that the poster campaign affected attitudes of mental health professionals towards suicidal patients, but only for those professionals that were assigned to the un-pretested condition (M = 12.0 vs. M = 12.6; t(14) = 2.58, *p* = 0.02). The corresponding between group effect size was 0.33.

## 4. Discussion

The present study was conducted to evaluate the impact of a brief educational suicide prevention poster campaign on knowledge, self-confidence and attitudes towards suicidal behaviour among staff of emergency and psychiatric departments. The educational poster and accompanying triage guide appears to have no effect on knowledge about suicide and self-confidence in suicide care in the studied population. However, the findings demonstrate that the poster campaign may positively affect attitudes, that is, lead to a greater willingness to help suicidal patients among staff of psychiatric departments.

The results do not accord with what was expected based on the evaluation of the SPRC “Is Your Patient Suicidal?” poster campaign in the United States [3], as less powerful evidence for the effectiveness of the educational poster campaign for health care professionals was found in Flanders. In the United States, approximately half of the ED staff members exposed to the poster and guide reported improvements in their self-perceived knowledge and skills regarding detection and treatment of suicidality, although it must be added that the US study suffered from some methodological shortcomings that could interfere with the interpretation of findings.

A possible explanation for the lack of effect of the poster campaign on knowledge levels can be found in the fact that in Flanders staff’s knowledge scores were already near its maximum at the start of the study. More specifically, almost half of the study group perceives their general understanding about suicide and suicide prevention as good or very good. High baseline levels regarding risk factors and warning signs are also observed, indicating that self-perceived knowledge level appears to reflect actual knowledge levels. Due to this pre-study knowledge, participants may experience a ceiling effect, making it more difficult to increase their knowledge about suicide and suicide prevention any further.

At baseline, clinical staff report not only a high level of knowledge, but also express a high level of willingness to help suicidal patients. A possible explanation can be found in the reliance on voluntary participation and, as a consequence, a relative homogenous sample that may have skewed the findings to be more positive than they otherwise might have been.

Further, at pre-test, the vast majority of mental health professionals perceive themselves as highly skilled in dealing with suicidal patients as they report no hesitancy in asking about patients’ suicidality and feel (very) confident in their ability to successfully detect and manage suicidal behaviour. A possible explanation can be found in the high rate of additional education or training in suicide prevention that staff of psychiatric departments received within 6 months prior to the study, which also may explain the high knowledge level at baseline. Compared to staff of psychiatric departments, ED staff report less confidence in identifying and intervening with suicidal patients and more hesitancy in discussing current suicidal ideation. This may be due to the low rate of education or training in suicide prevention among this occupational group, as only one in three ED providers were trained or educated in suicide prevention in the 6 months prior to the baseline assessment.

### 4.1. Strengths and Limitations

This study provides insights into the effect of suicide prevention poster campaigns as there is a clear lack of scientifically sound evaluation of suicide prevention strategies in terms of early detection of suicide risk, intervention, follow-up and referral of suicidal patients. The clustered randomized controlled Solomon four-group design is rare in this field of research, but most certainly represents a strength of this study. A cluster randomized controlled trial of this size provides a large amount of evidence.

The present study contributes meaningfully to the understanding of the effectiveness of an educational suicide prevention poster campaign as a small significant effect on attitudes of mental health care providers could be observed. However, this result should be interpreted with caution due to the increased chance of a type I error caused by the high number of comparisons made associated with the Solomon four-group design.

Possible reasons for the little effect of the poster campaign may be lack of simple design impact, content and location of the posters due to information overload that often characterizes the clinical workspace.

Several occupational groups were underrepresented in the study, such as physicians and psychiatrists. It is recommended to evaluate the effect of this poster as a part of a multimodal educational programme in a more heterogeneous sample thus targeting other gatekeepers as well.

### 4.2. Implications for Practice and Directions for Future Research

In this trial, the use of an educational poster campaign does not lead to improved knowledge and self-confidence and has little beneficial impact on attitudes of studied health care providers, most probably due to high levels of pre-study knowledge and experience. However, a poster campaign may be an effective tool in raising awareness when embedded in a broader suicide prevention strategy. In addition, it is recommended that pre-study knowledge is assessed and that preventive efforts using a poster particularly target care providers with limited knowledge.

Advancing training to detect, intervene and follow-up individuals at risk for suicide is widely recommended as suicide prevention policy for all health professionals and gatekeepers. Therefore, the poster and accompanying triage guide will be used as an additional tool as part of broader suicide prevention training programmes that are provided in Flemish hospitals.

## 5. Conclusions

This randomised controlled trial provides limited evidence for the effectiveness of an educational poster campaign for suicide prevention. As the studied population appears to have relatively high baseline knowledge about suicide, further research is needed in a more heterogeneous sample targeting other gatekeepers in various health domains with limited knowledge as well. Furthermore, it is recommended to evaluate the effect of this poster and accompanying triage guide as a part of a multimodal educational programme.

## Figures and Tables

**Table 1:** Baseline characteristics of the intervention and control group at the cluster level and the individual level in *n* (%) unless otherwise stated. (Totals do not always equal 212 (intervention group) or 338 (control group) due to missing data.)

**Department Level**		
	**Intervention Group** **(Pre-Test Condition)** **(*n* = 14)**	**Control Group** **(Pre-Test Condition)** **(*n* = 22)**
**Cluster Size**	Mean = 15.1	Mean = 15.4
	Median = 18	Median = 22
	Min = 4 Max = 22	Min = 4 Max = 29
**Department Type**		
emergency department	7 (50.0)	11 (50.0)
psychiatric department	7 (50.0)	11 (50.0)
**Staff Level**		
	**Intervention Group** **(*n* = 212)**	**Control Group** **(*n* = 338)**
**Gender**		
men	68 (33.2)	89 (28.7)
women	137 (66.8)	221 (71.3)
**Age**		
18–25 years	32 (15.7)	42 (13.5)
26–35 years	64 (31.4)	107 (34.5)
36–45 years	43 (21.1)	73 (23.5)
46–55 years	50 (24.5)	62 (20.0)
56–65 years	15 (7.4)	26 (8.4)
mean (SD) years	38.3 (11.4)	38.2 (11.0)
**Department Type**		
emergency department	88 (41.5)	155 (45.9)
psychiatric department	124 (58.5)	183 (54.1)
**Professional Discipline**		
physicians	5 (2.4)	10 (3.2)
psychiatrists	6 (2.9)	10 (3.2)
psychologists	9 (4.4)	11 (3.5)
psychiatric nurses	60 (29.3)	75 (24.2)
nurses	102 (49.8)	167 (53.9)
social workers	5 (2.4)	10 (3.2)
paramedics	11 (5.4)	14 (4.5)
other	7 (3.4)	13 (4.2)
**Experience**		
practice experience mean (SD) years	15.0 (10.7)	14.2 (10.5)
experience with suicidal behaviour		
daily	126 (40.9)	95 (46.8)
once a week	113 (36.7)	73 (36.0)
once a month	40 (13.0)	20 (9.9)
4–5 times a year	23 (7.5)	11 (5.4)
once a year	3 (1.0)	3 (1.5)
never	3 (1.0)	3 (1.5)
**Outcome Measures**		
self-evaluation of knowledge mean (SD)	23.8 (3.8)	24.3 (4.0)
actual knowledge regarding risk factors mean (SD)	2.2 (0.7)	2.1 (0.8)
actual knowledge regarding warning signs mean (SD)	4.0 (0.9)	3.9 (0.9)
provider confidence mean (SD)	11.2 (2.1)	10.8 (2.2)
helping attitudes towards suicidal patients (SD)	9.9 (1.5)	9.7 (1.6)

## Supplementary Materials

The following are available online at www.mdpi.com/1660-4601/14/3/304/s1, Questionnaire S1: SHORT SURVEY ON KNOWLEDGE, SELF-CONFIDENCE AND ATTITUDES TOWARDS SUICIDAL BEHAVIOUR.
