# Supplementary material for: Knowledge, Self-Confidence and Attitudes towards Suicidal Patients at Emergency and Psychiatric Departments: A Randomised Controlled Trial of the Effects of an Educational Poster Campaign
Source: Int J Environ Res Public Health. 2017 Mar 14;14(3):304. doi: 10.3390/ijerph14030304 (PMC5369140; doi:10.3390/ijerph14030304)
Supplement: Supplementary file 1 [file ijerph-14-00304-s001.pdf]

SHORT SURVEY ON KNOWLEDGE, SELF-CONFIDENCE AND ATTITUDES TOWARDS SUICIDAL BEHAVIOUR

Please answer all questions one by one in sequence. Always tick the checkbox that best applies to you. Thank you very much.

Knowledge	very low	low	neutral	high	very high
1.1. Facts about suicide	☐	☐	☐	☐	☐
1.2. Suicide warning signs	☐	☐	☐	☐	☐
1.3. How to ask someone who may be suicidal	☐	☐	☐	☐	☐
1.4. Persuading someone to get help	☐	☐	☐	☐	☐
1.5. How to get help for someone who may be suicidal	☐	☐	☐	☐	☐
1.6. Information about local resources for help with suicide	☐	☐	☐	☐	☐
1.7. Please rate your general understanding about suicide and suicide prevention	☐	☐	☐	☐	☐

	agree	disagree
2.1. Suicide is always the act of a mentally ill or psychotic person.	☐	☐
2.2. There is no relationship between drugs/alcohol and suicide.	☐	☐
2.3. There are one or two causes or motives that explain most suicides.	☐	☐
2.4. Removing the means of suicide (e.g., removal of handguns and poisons) would prevent many suicides.	☐	☐
2.5. Suicidal tendencies are inherited, and suicide runs in families.	☐	☐

2.6. Everyone who commits suicide is depressed.				☐	☐
2.7. People who make plans to commit suicide keep their thoughts to themselves and the suicide occurs without warning.				☐	☐
2.8. People who talk about suicide do not kill themselves.				☐	☐
**Self-confidence**	**strongly disagree**	**disagree**	**undecided**	**agree**	**strongly agree**
3.1. I am confident in my ability to successfully assess suicidal patients.	☐	☐	☐	☐	☐
3.2. I am confident in my ability to successfully treat suicidal patients.	☐	☐	☐	☐	☐
3.3. I am hesitant to ask a patient if he or she is suicidal.	☐	☐	☐	☐	☐
**Attitudes**	**completely disagree**	**disagree**	**undecided**	**agree**	**completely agree**
4.1. It is a human duty to try to stop someone from dying by suicide.	☐	☐	☐	☐	☐
4.2. If someone wants to commit suicide, it is their business and we should not interfere.	☐	☐	☐	☐	☐
4.3. I am prepared to help a person in a suicidal crisis by making contact.	☐	☐	☐	☐	☐

Thank you for your cooperation!
